# Emphysematous Gastritis in a Chronic Alcohol User: A Case of Gastric Pneumatosis With Suspected Infectious Etiology

**DOI:** 10.7759/cureus.90360

**Published:** 2025-08-18

**Authors:** Shamsun Nahar, Eric Field, Rokeya Begum, Nipa Islam, Nahid Hasan

**Affiliations:** 1 Internal Medicine, California Institute of Behavioral Neurosciences and Psychology, Fairfield, USA; 2 Hospital Medicine, Augusta Health, Fisherville, USA; 3 Internal Medicine, Jalalabad Ragib Rabeya Medical College, Sylhet, BGD; 4 Internal Medicine, Dhaka Medical College, Dhaka, BGD; 5 Internal Medicine, Institute of Applied Health Science (IAHS), Dallas, USA

**Keywords:** alcohol, emphysematous gastritis, empiric treatment, gastric emphysema, infectious etiology

## Abstract

Emphysematous gastritis (EG) is a rare but potentially fatal form of gastric pneumatosis caused by gas-forming organisms infiltrating the gastric wall, often in the context of mucosal injury or systemic vulnerability such as chronic alcohol use. We present the case of a 55-year-old male with a history of daily alcohol consumption who arrived with severe epigastric pain and over 100 episodes of vomiting in a single day. Laboratory findings revealed leukocytosis, electrolyte imbalances, and acute kidney injury, while CT imaging showed gas within the gastric wall, consistent with EG. The patient responded favorably to conservative management, including intravenous antibiotics, proton pump inhibitors, fluid resuscitation, and symptomatic care. Although microbiological confirmation was not obtained, the clinical course strongly suggested an infectious etiology. This case highlights the importance of distinguishing EG from gastric emphysema, initiating early empirical therapy, and addressing underlying risk factors such as alcohol use to improve outcomes.

## Introduction

Gastric pneumatosis, the presence of air within the gastric wall, is a rare finding that has been recognized both clinically and radiologically for over a century [1-3]. This condition occurs due to disruption of the gastric wall integrity, which can be caused by numerous etiologies, such as infection, pneumothorax, instrumentation, gastric wall ischemia, mechanical injury from excessive retching, vomiting, gastric outlet obstruction (secondary to pyloric stenosis or carcinoma), and others [1,4]. Emphysematous gastritis (EG) is mostly infectious in etiology, particularly by gas-forming bacteria that form gas bubbles, while gastric emphysema (GE) is mostly non-infectious [5,6]. It is pivotal to distinguish between EG and GE due to the high mortality rate associated with the former [1]. In this case study, we describe a middle-aged male with chronic alcohol consumption who had a history of nausea and vomiting, as well as gastric pneumatosis discovered by CT scan. We will concentrate on identifying the differences between EG and GE from the infectious disease viewpoint.

## Case presentation

A 55-year-old male arrived at the emergency room with about a hundred episodes of nausea and vomiting that began early on the day of admission. The vomitus was made up of stomach contents devoid of coffee grounds or blood. In addition, he experienced a maximum intensity of 8/10 for cramping, burning, and sporadic severe epigastric pain that radiated to the chest and abdomen and was made worse by vomiting. The patient denied experiencing any fever, blood in the stool, or chest pain. But he reported periodic chills, diaphoresis, and loose stools. Following a similar event three months prior, the patient was diagnosed with acute pancreatitis without any visible signs of necrosis or infection. Social history was positive for cigarette smoking, marijuana use, and alcohol consumption between half to one pint each day.

Despite being awake and oriented, the patient seemed to be unwell. When he was admitted, he had a normal temperature, a pulse of 76, a respiratory rate of 18 breaths per minute, and a blood pressure of 153/98 mmHg. Upon examination, he had dry mucous membranes, and his epigastric region showed minor pain to the touch and voluntary guarding. There was no evidence of rebound tenderness. It was a soft, nondistended abdomen; there were bowel sounds throughout.

On admission, the patient demonstrated leukocytosis, with a white blood cell count elevated to 11.3 ×10³/µL. Electrolyte panel revealed hypokalemia, with a potassium level of 3.0 mmol/L, and hyponatremia with a sodium concentration of 131 mmol/L. Liver function tests were abnormal, showing an alkaline phosphatase level of 201 IU/L, alanine transaminase (ALT) of 88 IU/L, and aspartate transaminase (AST) of 187 IU/L, consistent with hepatic injury. Lipase was within normal limits at 27 IU/L. Renal function was impaired, with a serum creatinine level of 1.37 mg/dL, elevated from a baseline of 1.02 mg/dL, meeting the Kidney Disease: Improving Global Outcomes (KDIGO) definition of Acute Kidney Injury (AKI). Table 1 summarizes the laboratory findings on admission, alongside normal ranges.

**Table 1:** Laboratory results upon admission with normal ranges and interpretation Data obtained with permission from lab results performed at the admitting hospital

Parameter	Measured Value	Normal Range	Interpretation
White Blood Cells	11.3 ×10³/µL	4.0–10.0 ×10³/µL	Elevated (Leukocytosis)
Potassium	3.0 mmol/L	3.5–5.1 mmol/L	Low (Hypokalemia)
Sodium	131 mmol/L	135–145 mmol/L	Low (Hyponatremia)
Alkaline Phosphatase	201 IU/L	44–147 IU/L	Elevated
ALT	88 IU/L	7–56 IU/L	Elevated
AST	187 IU/L	10–40 IU/L	Elevated
Lipase	27 IU/L	10–140 IU/L	Normal
Creatinine	1.37 mg/dL	0.6–1.3 mg/dL	Elevated

Imaging Studies

A computed tomography (CT) scan of the abdomen/pelvis performed during admission revealed moderate distention of a fluid-filled stomach with the presence of air within the wall of the stomach, consistent with gastric pneumatosis. Additional findings included a hiatal hernia, fatty infiltration of the liver, and non-visualization of the appendix, suggesting no acute appendicitis. The pancreas, kidneys, and ureters appeared normal. The chest X-ray was unremarkable, and an ECG revealed a normal sinus rhythm with no acute changes.

**Figure 1:**
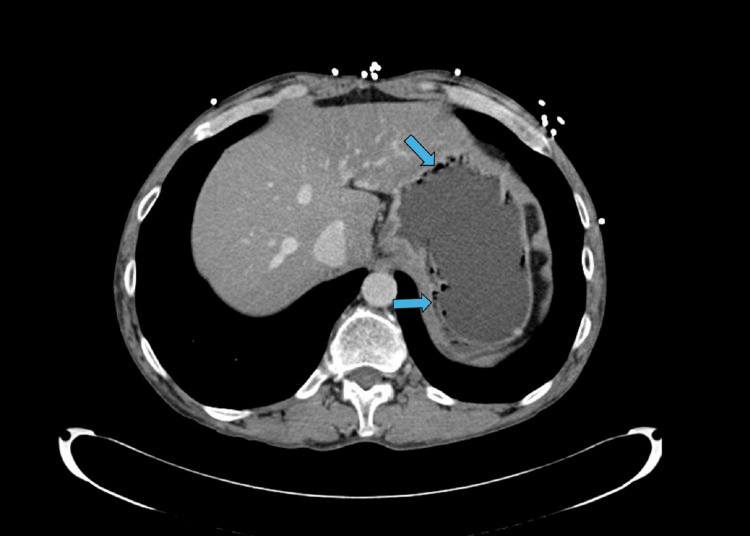
Computed tomography scan of the abdomen (axial view) The image shows marked distention of the fluid-filled stomach. Blue arrows indicate tracking along the stomach concerning gastric pneumatosis.

**Figure 2:**
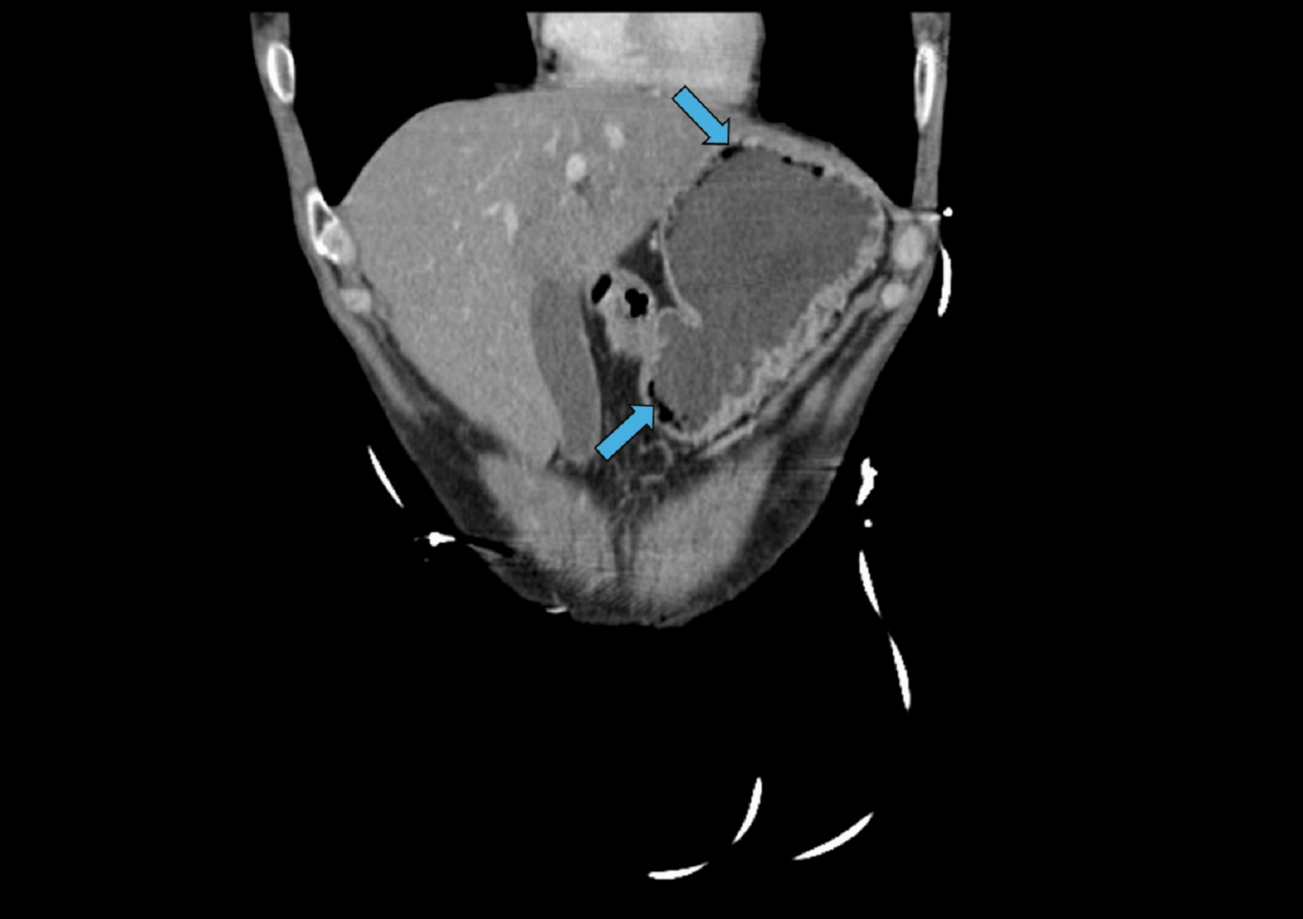
Computed tomography scan (coronal view) Blue arrows indicate the presence of gas within the stomach wall.

The following two images show CT abdomen pelvis findings. Figure 1 shows the axial view of the CT. There is marked distention of the fluid-filled stomach and tracking along the wall of the stomach concerning gastric pneumatosis, marked by arrows. Figure 2 shows the coronal view, with the presence of gas within the stomach wall marked by arrows.

Given the patient’s presentation with severe epigastric pain, nausea, vomiting, and leukocytosis, several differential diagnoses were considered. Acute pancreatitis was initially suspected due to the patient’s prior history; however, normal lipase levels and unremarkable pancreatic imaging ruled this out. Peptic ulcer disease and gastrointestinal bleeding were excluded based on the absence of hematemesis, melena, or coffee-ground emesis, and no evidence of mucosal disruption on imaging. Gastroesophageal reflux disease (GERD) and hiatal hernia were considered but deemed less likely due to the severity of symptoms and radiologic findings. Gastric outlet obstruction was excluded based on the absence of persistent vomiting of undigested food and normal bowel sounds. Finally, gastric emphysema was considered a benign mimic of emphysematous gastritis; however, the presence of systemic signs (leukocytosis, elevated liver enzymes, AKI), imaging findings of gas within the stomach wall (shown in Figures 1, 2), and clinical improvement with antibiotics supported the diagnosis of emphysematous gastritis over gastric emphysema.

Upon consultation, both gastroenterology and surgery suggested conservative therapy, as there was currently no justification for surgical intervention. The hepatitis panel was checked per gastroenterology recommendation, which later came out negative. We started administering intravenous (IV) pantoprazole 40 mg twice daily, piperacillin/tazobactam antibiotics because of the suspected infectious gastritis, and lactated Ringer's fluids to help with dehydration. Because of the intensity of nausea and vomiting, supportive care and ondansetron symptomatic therapy continued. The patient had low potassium and sodium levels, indicators of dehydration. Probably secondary to dehydration-related prerenal factors, he had AKI with elevated creatinine of 1.37 (baseline 1.02), but these improved after IV hydration to 0.86 and 135, respectively. He was supplemented with potassium as required. Close attention was paid to electrolytes and renal function.

He was started on multivitamins for alcohol use, such as thiamine and folic acid, and the Clinical Institute Withdrawal Assessment for Alcohol (CIWA) protocol. Patient's maximum CIWA score was 09. He was also prescribed lorazepam to treat withdrawal symptoms and nicotine replacement therapy for tobacco use.

The patient's nausea and vomiting subsided with conservative treatment, and he was switched to a clear liquid diet on the following day. He was able to handle the diet, and his electrolytes and renal function held steady. His WBC was improved to 6.8. Although his initial leukocytosis could be stress-related, considering the suspected pathophysiology and prompt response to antibiotics indicates a more infectious etiology. There were no symptoms of alcohol withdrawal. Thereafter, the patient returned to a regular diet, which he managed to endure without experiencing severe discomfort or vomiting. Upon his release, he was prescribed oral pantoprazole, potassium supplements, antibiotics (augmentin), and antiemetics as needed. A bland diet was advised for a week, followed by a follow-up visit with the primary care physician for additional electrolyte monitoring in a week and gastroenterology in two weeks for continued treatment of alcohol-related gastritis and potential alcohol cessation techniques. Additionally, outpatient substance abuse counseling was advised for alcohol, tobacco, and marijuana use.

## Discussion

Definition and classification

The air inside the stomach wall is generally referred to as gastric pneumatosis, and it falls into two categories: gastric emphysema (GE) and emphysematous gastritis (EG). Both can present similarly clinically and radiologically, but EG has a worse prognosis, and GE is self-limiting with benign results [2,6]. GE is further subclassified as pulmonary, obstructive, and traumatic [4,7,8].

Pathophysiology and risk factors

The pathophysiology and risk factors of gastric pneumatosis are diverse and can occur in a range of clinical scenarios, from benign to fatal, such as ischemia from gas-producing organisms (EG), infarctions, or increased intraluminal pressure [9]. Although imaging is the primary diagnostic method, its ability to reveal the underlying cause or prognosis is questionable [6,10,11].

Tables 2, 3 below explain the pathophysiology of gastric pneumatosis. Table 2 shows etiologies of gastric pneumatosis by mechanism. Table 3 summarizes the pathophysiology and predisposing factors of emphysematous gastritis. There are two major categories: GE and EG. Within GE, there are three subcategories: pulmonary, obstructive, and traumatic. In addition to these categories, ischemia or infarction of the colon or small bowel can cause air entry into the stomach. The authors created the tables by utilizing information gathered from the citations listed in the footnotes.

**Table 2:** Etiologies of gastric pneumatosis by mechanism GE: Gastric emphysema. The table was generated using data from [4,5,7–11].

Mechanism	Description	Common etiologies
Traumatic	Physical damage or tear of the gastric mucosa	Gastrostomies, endoscopies, nasogastric tube placements etc.
Obstructive	Mucosal tear due to distension and luminal air diffusion from distal obstruction	Peptic ulcer disease, Carcinoma, duodenal obstruction, pyloric stenosis, Superior mesenteric artery syndrome (SMAS), gastric volvulus etc.
Pulmonary	Mediastinal air penetration into the gastric wall due to increased intrapulmonary pressure	Chronic obstructive pulmonary disease (COPD), Asthma, alveolar rupture, pneumothorax etc.

**Table 3:** Pathophysiology and predisposing factors of emphysematous gastritis EG: Emphysematous gastritis. The table was generated using data from [1,4,5,7–11].

Category	Details
Pathophysiology	Infection by a gas-forming organism in the stomach wall, which produces and accumulates gas as its metabolic byproduct
Predisposing Factors	Ingestion of corrosive substances, alcoholism, malnutrition, diabetes mellitus, renal failure, usage of Non-Steroidal Anti-Inflammatory Drugs (NSAID), recent abdominal surgery. gastroenteritis
Common Organisms	Klebsiella pneumoniae, Escherichia coli, Enterobacter spp, Pseudomonas aeruginosa, Clostridium welchii, Staphylococcus aureus, Bacillus subtilis, Bacillus proteus, Candida spp

Besides these, Garrosa-Munoz et al. reported the first case report on suspected SARS-CoV-2-associated EG because of the ability of SARS-CoV-2 to invade the gastrointestinal Angiotensin Converting Enzyme 2 (ACE2) receptor and hypercoagulability due to endothelial cell injury. However, because it was the first case, they suggested more research on that to be done in the future [12].

Our patient's pathology was influenced by several conditions mentioned above, including acute vomiting and drunkenness. Although not microbiologically verified, our patient's drunkenness likely contributed to an infectious etiology of EG as well. While the patient is predisposed to both EG and GE due to prolonged alcoholism, gas-forming organisms are mostly responsible for EG, given his symptoms of nausea, vomiting, and abdominal pain.

We empirically started our patient on Zosyn, which covers methicillin-susceptible *Staphylococcus aureus*, *Klebsiella pneumoniae*, *Escherichia coli*, Enterobacter species, *Pseudomonas aeruginosa*, and *Clostridium welchii*. Nevertheless, it excludes Candida, *Bacillus subtilis*, and Bacillus proteus species. Therefore, we empirically addressed the bulk of EG's infectious etiologies.

Presentation

While both GE and EG can radiologically reveal gas in the stomach wall, GE may show up with little to no discomfort or without any symptoms. In addition to more serious gastrointestinal symptoms such as nausea, vomiting, and abdominal pain, EG frequently manifests with inflammatory symptoms like fever, tachycardia, vomiting, leukocytosis, and the presence of air in the stomach wall [7]. Sepsis or septic shock may also accompany EG [11,13]. However, both may have comparable symptoms. We suspect the patient had EG rather than GE, and the origin of EG is infectious because our patient experienced leukocytosis, gas in the stomach wall on the CT scan, severe nausea, vomiting, and abdominal discomfort.

Treatment

Guidelines for managing gastric pneumatosis are not standardized. Nothing per mouth, intravenous fluids, and monitoring are the conservative methods to treat GE. EG is actively handled because of its high mortality rate [1]. If conservative care fails, EG is aggressively treated with antibiotics, intravenous hydration, pressor support, bowel rest, proton pump inhibitors (PPI), and, if necessary, urgent laparotomy or gastrectomy [13]. We effectively treated the patient using a more cautious approach, including PPI, bowel rest, IV antibiotics, and surgery and GI consultation, which yielded no surgical indications.

Prognosis and complications

EG has remarkable clinical symptoms and a mortality rate of about 60%, whereas GE is primarily self-limited. The mortality rate has been cut in half because of a more current treatment strategy [3,11]. If a gas-producing bacterium brings on emphysematous gastritis, the prognosis is worse, particularly when portal gas is present [14]. Gastric contractures and fibrosis are the most frequent long-term problems [11].

## Conclusions

This case report emphasizes the significance of promptly diagnosing and treating emphysematous gastritis, particularly in long-term alcohol users who are more susceptible to infectious consequences. While EG can have a worse prognosis and necessitate extensive therapy, GE is benign. Based on clinical suspicion of infectious pathology, we started our patient on empirical antibiotics that target common bacteria, and this led to a notable improvement. The patient's reaction to treatment points to a potential infectious pathology, even if microbiological confirmation was not established. Our patient's positive outcome demonstrates the value of early treatment and alcohol cessation counseling in preventing recurrence.
